# Effect of diabetes mellitus on survival in patients with gallbladder Cancer: a systematic review and meta-analysis

**DOI:** 10.1186/s12885-020-07139-y

**Published:** 2020-07-24

**Authors:** Chen Jing, Zhengyi Wang, Xue Fu

**Affiliations:** School of Nursing and Health, Nanfang College of Sun Yat-sen University, Guangzhou, 510970 Guangdong Province China

**Keywords:** Gallbladder cancer, Diabetes mellitus, Mortality, Meta-analysis

## Abstract

**Background:**

Increasing evidences indicated that diabetes might increase the incidence of gallbladder cancer. However, no sufficient data has ever clarified the impact of diabetes on the survival of patients with gallbladder cancer.

**Methods:**

We comprehensively searched PubMed, Embase, and the Cochrane Library databases through July 2019 in order to find sufficient eligible researches. The pooled hazard risks (HRs) and relative risks (RRs) with 95% confidence intervals (CIs) were calculated with either fix-effects or random-effects model. Due to the low gallbladder cancer mortality in general population, the RRs and standard mortality ratios (SMRs) were considered the similar estimates of the HRs.

**Results:**

Ten eligible studies were included in this meta-analysis. Analysis of eight cohorts found that diabetes was closely associated with the mortality of gallbladder cancer (HR = 1.10; 95% CI: 1.06–1.14; *P* < 0.00001). However, the mortality in male diabetes patients was not higher than female patients (RR = 1.08, 95%CI = 0.57–2.04, *P* = 0.80).

**Conclusions:**

These findings indicated that diabetes patients had a higher mortality of gallbladder cancer compared with non-diabetes.

## Background

Gallbladder cancer (GBC) is one of the most common biliary tract malignancies worldwide [1]. By and large, poor prognosis seriously affects the mortality of patients with gallbladder cancer [2]. Gallbladder cancer patients survive the mean survival rate of 6 months and a 5-year survival rate of 5% [3]. Generally, women are two to six times more likely to be attacked by gallbladder cancer [4]. The prognosis of patients with GBC is affected by a growing number of factors, including age, gender, smoking, ethnic, and menopause [5–9]. Advancing age partly demonstrates the prevalence of gallbladder cancer [10]. Finding an optimal prognostic indicator would be helpful to improve the survival rate of GBC.

Diabetes mellitus (DM) is a costly chronic disease worldwide. The incremental increase in costs of this disease have laid economic burdens on both financial expenditure in most countries and patients themselves. In the United State, the newly diagnosed patients spent approximately $8941 more than subjects who were not diagnosed with DM over a period of 5 years [11]. Approximately 415 million people suffered from diabetes in 2015 while 5 million patients died from diabetes [12]. By 2040, the number of diabetes patients are predicted to ascend to 642 million. DM is always regarded as a pivotal risk factor linked to cancer at different sites, including lung [13], liver [14], esophagus [15], stomach [16], colorectum [17], kidney [18], breast [19], leukemia, non-Hodgkin lymphoma, myeloma [20], ovary [21], and prostate [22]. As several studies and meta-analyses have pointed out, DM was closely associated with the onset risk of gallbladder cancer [23, 24]. However, rare study has focused on the relationship between DM and the mortality of gallbladder cancer. This meta-analysis aimed to figure out if DM patients had a higher risk of dying from GBC and if male and female patients had a different risk of die from GBC.

## Methods

### Search strategy

A comprehensive search has been made on the PubMed, Embase, web of science, and the Cochrane Library databases to find all the eligible studies up to July 13th 2019. The following text words were used in the PubMed: (“diabetes” OR “glucose intolerance” OR “insulin resistance” OR “hyperglycemia” OR “hyperinsulinemia” OR “metabolic syndrome”) AND (“gallbladder cancer” OR “gallbladder carcinoma” OR “gall bladder cancer” OR “gall bladder carcinoma”). Correlative key words were used in the Embase, web of secience, and the Cochrane Library. To comprehensively search eligible studies, we simultaneously searched the reference lists of relevant reviews or included publications for further studies.

### Inclusion and exclusion criteria

The included literatures met the following criteria: (1) cohort design; (2) investigated gallbladder cancer outcomes; (3) assessed the gallbladder cancer mortality with or without DM; (4) reported the information of hazard ratios (HRs), relative risks (RRs), or standard mortality ratios (SMRs). The exclusion criteria were as follows: (1) case-control or cross-sectional design; (2) unavailable data.

### Data extraction

Two authors independently extracted all data from publications using the same criteria. The following data were included: the first author’s name, publication year, country, sample size, the number of male or female participants, mean age at baseline, average follow-up duration, diabetes assessment, and adjusted factors.

### Statistical analysis

We used Reviewer Manager 5.3 in this meta-analysis to analyze the data. The pooled HRs with 95% CIs were calculated as the effect estimates for the relationship between DM and gallbladder cancer mortality. The fixed-model was used when the heterogeneity was low, while the random-model was used when the heterogeneity was high. Owing to the low gallbladder cancer mortality in general population, the RRs, SMR were considered the similar estimates of the HRs [25]. Statistical heterogeneity among studies was assessed by the I^2^ and Q statistics. Both I^2^ > 50% and *P* value< 0.1 were regarded as high heterogeneity. We conducted subgroup analysis to evaluate the potential sources of heterogeneity from country, follow-up duration, diabetes assessment, and adjusted factors (including BMI, smoking, and education). A sensitivity analysis was performed by removing each study from the overall analysis to investigate the influence of a single study. We used funnel plots, Begg and Egger tests to assess publication bias. *P* value< 0.05 was viewed as a significant level. The statistical analyses were performed with Stata software (version 12.0).

## Results

### Study selection

**Fig. 1:**
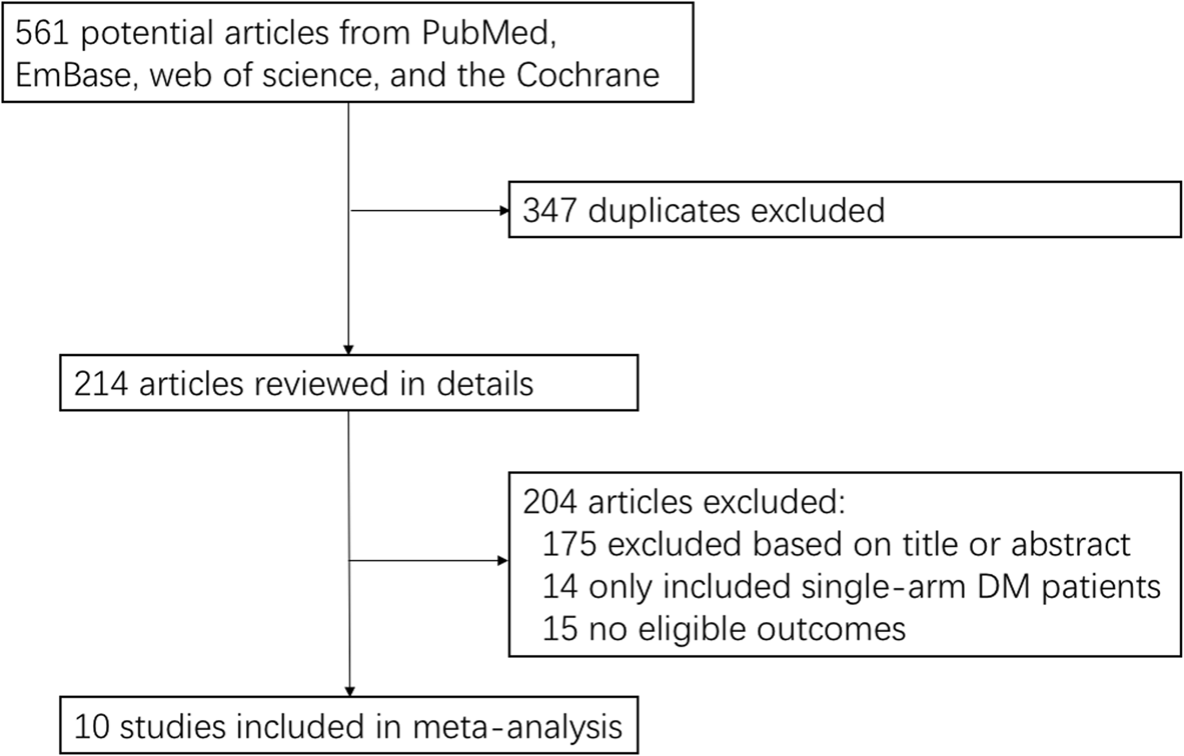
Flow-chart of study selection for the meta-analysis

Detailed study selection process was described in Fig. 1. From the initial search, we searched and identified 561 records. Two authors independently assessed the search outputs based on the primary research title or abstract. Three hundred forty-seven articles were discarded for the sake of duplication. One hundred seventy-five articles were excluded based on title or abstract. Then we read the full-text of the remaining paper. We further removed 14 studies that enrolled single-arm DM patients. Fifteen of the 25 remaining studies were subsequently removed due to lack of eligible data. Finally, a total of 10 studies were included in the meta-analysis [26–35].

### Study characteristics

The baseline characteristics of the included studies were listed in Table 1. A total of 5,522,636 participants were included in all 10 studies. Two studies were conducted in the USA, two in the UK, three in the Asia, one in Australia, and two were international conducted studies. The average follow-up duration ranged from 2 to 18 years. Diabetes assessment methods included self-report, medical record, WHO diagnostic criteria, and read code classification. Eight studies reported the relationship between DM and gallbladder cancer mortality, while four studies assessed the different gallbladder cancer mortality in male and female DM patients.

**Table 1:** Characteristic of studies included in the meta-analysis

First author, publication year	Country	Sample size	Male/female	Mean age (year)	Average follow-up duration (year)	Effect measure	Diabetes assessment	Adjusted factors
Coughlin, 2004 [26]	USA	1,056,243	467,922/588321	56.7	12.5	RR	Self-report	Age, smoking, race, BMI, exercise, education
Yagyu, 2004 [27]	Japan	113,394	47,673/65721	40–89	9.7	HR	Self-report	Age, gender, history of hepatic disease
Swerdlow, 2005 [28]	UK	28,900	15,688/13212	NA	18.0	SMR	Medical record	Age, region, duration
Tseng, 2009 [34]	Taiwan	244,920	113,347/131573	NA	12	SMR	Medical record	Age, gender
Lam, 2011 [29]	Asia, Australia	367,361	216,743/150618	48	4	HR	Self-report or WHO diagnostic criteria	Age
Seshasai, 2011 [30]	Members of ERFC	820,900	426,868/394032	55	NA	HR	Medical record	Age, gender, smoking, BMI
Campbell, 2012 [35]	USA	1,053,831	467,143/586688	63.1	12.1	RR	Self-report	Age, BMI, education, exercise, NSAIDs, alchhol
Currie, 2012 [31]	UK	112,408	54,086/58322	67.8	2	HR	Read code classification	Age, gender, smoking, Charlson comorbidity index, year of diagnosis
Harding, 2015 [32]	Australia	953,382	506,312/447070	T1DM: 27.4 T2DM: 60.4	10	SMR	Medical record	Age
Chen, 2017 [33]	Asia	771,297	391,619/379678	53.9	12.7	HR	Self-report	Age, gender, BMI, smoking, alcohol, education, region

*ERFC* Emerging Risk Factors Collaboration, *T1DM* Type 1 Diabetes Mellitus, *T2DM* Type 2 Diabetes Mellitus, *RR* Relative Risk, *HR* Hazard Ratio, *SMR* Standard Mortality Ratio, *WHO* World Health Organization, *BMI* Body Mass Index, *NSAIDs* Nonsteroidal Anti-inflammatory Drugs

**Fig. 2:**
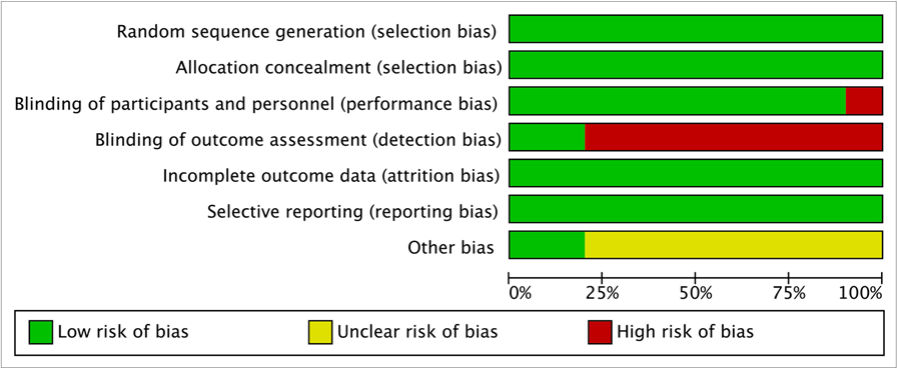
Overall risk of bias of the 10 included studies

**Fig. 3:**
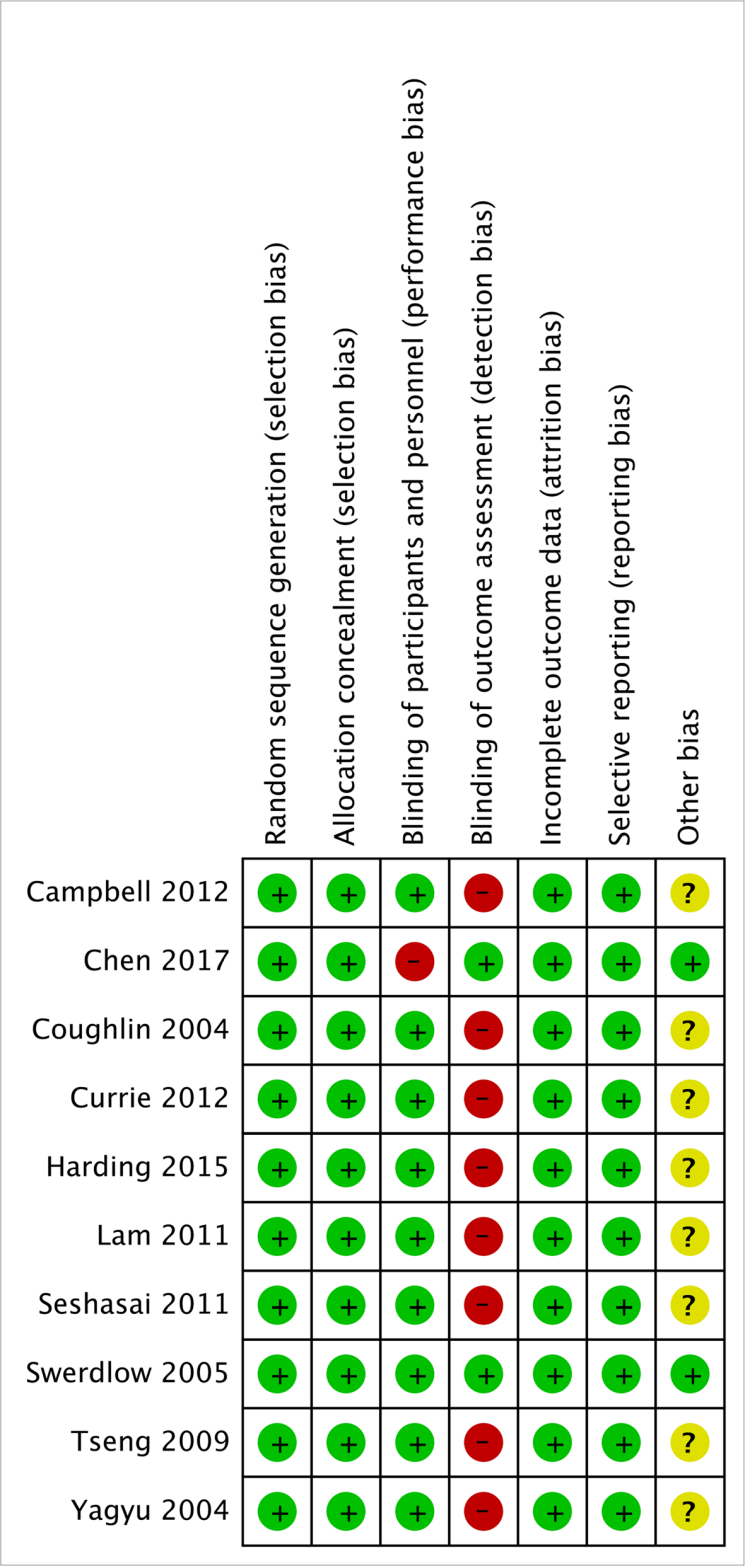
Risk of bias graph of the 10 included studies

The quality assessment results were shown in Figs. 2 and 3. All of the studies applied random sequence generation and allocation concealment. No attrition bias and reporting bias were reported. Two of all studies completed blinding of outcome assessment. Only one study reported performance bias.

### DM and gallbladder cancer mortality

**Fig. 4:**
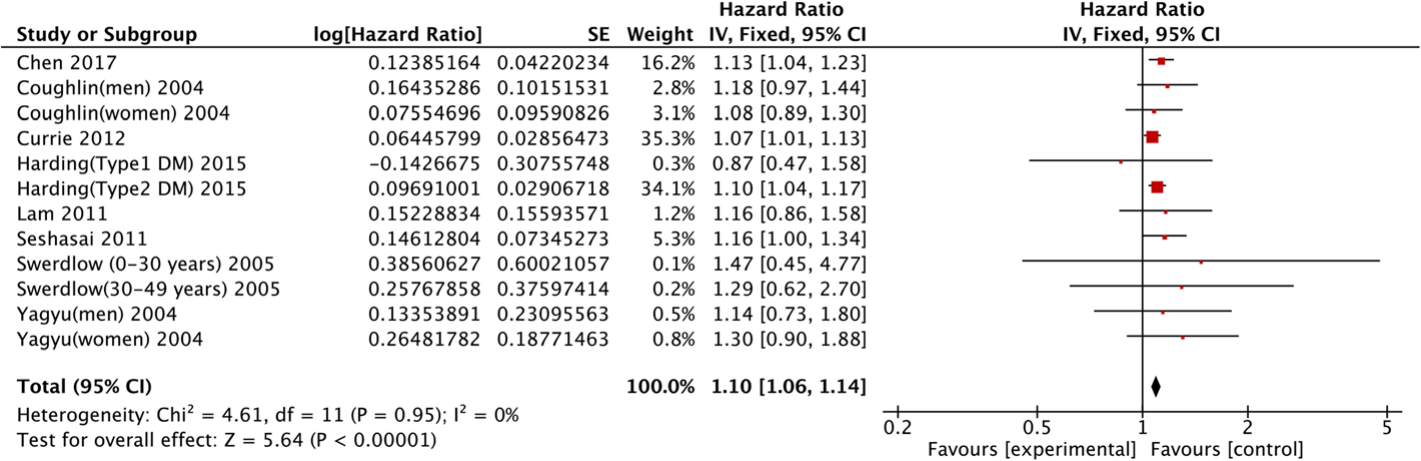
Association between diabetes mellitus and the mortality of gallbladder cancer

Eight studies focused on the relationship between diabetes mellitus and gallbladder cancer mortality. We merged the data of these studies and found that pre-existing diabetes had a high correlation with the mortality of gallbladder cancer compared with non-DM participants (HR = 1.10; 95% CI: 1.06–1.14; *P* < 0.00001; Fig. 4). A fix-effects model was applied owing to low heterogeneity (I^2^ = 0%; *P* = 0.95). The sensitivity analysis results indicated that the summary HR ranged from 1.09 (95%CI: 1.06–1.13) when excluding study from Chen 2017 to 1.12 (95%CI: 1.07–1.17) when excluding study from Currie 2012 [31, 33].

Subgroup analysis were conducted according to country, follow-up duration, diabetes assessment, and adjustment for confounding factors, including BMI, smoking, and education. All of the results were demonstrated in Table 2. However, no evidence indicated that there were significant differences between subgroups based on factors above.

**Table 2:** Subgroup analysis of relative risk for gallbladder cancer mortality in DM patients

Subgroup	No. of references	HR and 95% CI	P_a_	I^2^%	P_b_
Country					
Western countries	4	1.09 (1.05–1.13)	< 0.0001	0%	0.29
Eastern countries	2	1.10 (1.06–1.13)	0.001	0%	
Follow-up duration					
≦10	4	1.03 (0.89–1.20)	0.70	84%	0.27
> 10	3	1.13 (1.06–1.21)	0.0005	0%	
Diabetes assessment					
Self-report	3	1.14 (1.06–1.22)	0.0003	0%	0.34
Medical record	3	1.01 (0.81–1.27)	0.92	73%	
Adjusted BMI					
Yes	3	1.14 (1.07–1.21)	< 0.0001	0%	0.30
No	5	1.04 (0.90–1.21)	0.57	78%	
Adjusted smoking					
Yes	4	1.10 (1.05–1.14)	< 0.0001	0%	0.63
No	4	1.05 (0.87–1.26)	0.64	44%	
Adjusted education					
Yes	2	1.13 (1.05–1.21)	0.0007	0%	0.43
No	6	1.06 (0.93–1.21)	0.35	78%	

*P*_*a*_ P value for heterogeneity within subgroup, *P*_*b*_ P value for subgroup differences

### DM and gallbladder cancer mortality in men and women

**Fig. 5:**
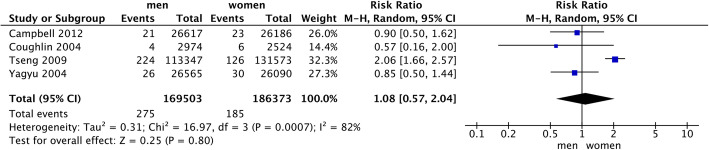
Different mortality of gallbladder cancer between male and female diabetes patients

A total of four studies estimated the difference of gallbladder cancer mortality between male and female DM patients. The analysis was conducted to see if female DM patients had a higher risk of gallbladder cancer mortality then male patients. The pooled analysis results demonstrated that no significant differences had existed between DM men and women (RR = 1.08, 95%CI = 0.57–2.04, *P* = 0.80; Fig. 5.). A random-effect model was applied due to high heterogeneity (*P* = 0.0007, I^2^ = 82%).

### Publication bias

**Fig. 6:**
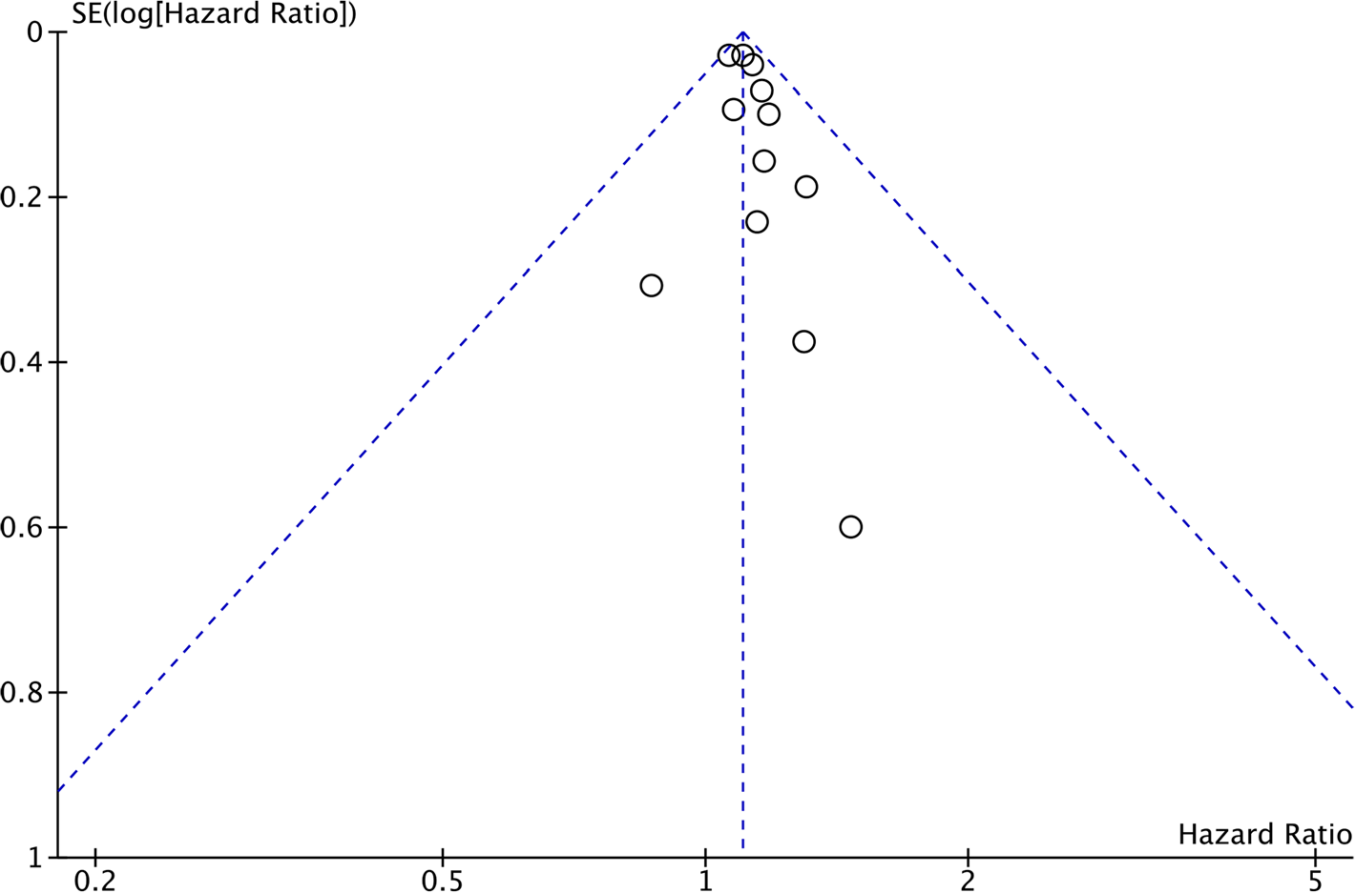
Funnel plot analysis of all the studies about the association between diabetes and gallbladder cancer

The symmetric funnel plots indicated a potential low publication bias (Fig. 6). Moreover, Egger test (*P* = 0.371) and Begg test (*P* = 0.845) showed no significant evidence of publication bias.

## Discussion

This meta-analysis of cohort studies provided comprehensive evidence that the diabetes mellitus had an impact on the survival of patients with gallbladder cancer. Our results suggested that diabetes patients had a higher mortality rate of gallbladder cancer compared with non-diabetes patients. And the results were independent of country, follow-up duration, diabetes assessment, BMI, smoking, or education. Though previous analysis had indicated that DM women were more likely to develop gallbladder cancer than DM men due to sex hormones [36], we found no obvious differences between male and female diabetes patients in gallbladder cancer mortality. However, the results remained to be tested due to lack of eligible data.

Several physiological mechanisms might account for the increase of gallbladder cancer mortality in DM patients. A growing number of studies have found that overweight, obesity, metabolic syndrome, and insulin resistance were closely related to the increase of gallbladder disease [37–39]. Hyperinsulinemia was also a phenomenon commonly existed in DM patients. Excess insulin directly or indirectly regulated the activity of insulin-like growth factor-1 (IGF-1), which was an important cytokine that influenced the development and progression of cancer [40]. Both in vitro and in vivo researches have proved that up-regulation of IGF-1 contributed to the proliferation of bile duct cancer cells and the inhibition of apoptosis [41, 42]. In addition, diabetes impaired the function of gallbladder emptying. The gallbladder smooth muscle cells of DM patients have reduced sensitivity to cholecystokinin. Meanwhile, the number of cholecystokinin receptors on the gallbladder wall in DM patients was also reduced [43]. These physiological mechanisms were consistent with the increased risk of biliary tract cancer [44].

To our knowledge, our meta-analysis was the first study focused on the impact of DM on the survival of patients with gallbladder cancer. Previous study has proved that diabetes might increase the risk of gallbladder diseases [45]. One meta-analysis has proved the association between DM and the increased GBC risk [24]. However, the meta-analysis included both case-control studies and cohort studies, which might somehow increase the overall heterogeneity. Furthermore, the majority of the included cohort studies focused on the gallbladder cancer incidence rather than mortality. Our analysis attempted to find an optimal prognostic indicator that would increase the GBC mortality. In addition, a subgroup analysis was conducted to see the difference of GBC mortality in male and female DM patients.

The present meta-analyses had some strengths, including prospective design of cohort studies, eligible data from large sample size, detailed subgroup analyses, and low heterogeneity. Our findings provided an important message for patients with comorbid DM and gallbladder cancer that preventing the progression of diabetes might increase the survival from gallbladder cancer.

There were several potential limitations in our study. First, residual confounding could not be ignored. Compared with non-DM participants, DM patients often had less healthy lifestyles, including higher rate of obesity, less physically activity, and more likely to smoke and drink. Though most of the included studies have adjusted these factors and our subgroup analysis showed no obvious heterogeneity between subgroups, we could not completely exclude the influence of these factors. Second, most studies did not tell the differences between type 1 and type 2 DM, though the majority of individuals were type 2 survivals. Older individuals were more likely to develop type 2 DM, while type 1 DM was a more common type in younger individuals. As a result of incomplete initial data on distinguishing this difference, some degree of inaccuracy of results was inevitable. Third, the number of eligible literatures remained low, which might have some influence on the final conclusion. The results of the difference of gallbladder cancer mortality between male and female patients remained open to question due to the lack of data and a high heterogeneity. Forth, the effect of medicine had not taken into account in the researches. Many studies have indicated that metformin, a commonly used diabetic medication, could retard the development of some cancers. None of the included researches have made adjustments for the use of diabetic medication. Last but not least, the multiplicity might exist in this analysis. The multiplicity attributed to a number of factors. On one hand, the subjects came from various backgrounds. Different rate, country, and age aggravated the multiplicity. On the other hand, the subjects from different studies might have an overlap.

## Conclusion

In conclusion, this meta-analysis suggested that diabetes patients had a higher mortality of gallbladder cancer. More relevant studies were needed to certify this association and tell the difference between men and women.

## Data Availability

All data generated in this analysis are available from the corresponding author.

## Abbreviations

HRs: Hazard risks
RRs: Relative risks
CIs: Confidence intervals
DM: Diabetes mellitus
ORs: Odd ratios
SMRs: Standard mortality ratios
BMI: Body mass index
IGF-1: Insulin-like growth factor-1
ERFC: Emerging Risk Factors Collaboration
T1DM: Type 1 Diabetes Mellitus
T2DM: Type 2 Diabetes Mellitus
WHO: World Health Organization
NSAIDs: Nonsteroidal Anti-inflammatory Drugs
